# Involvement of kinesins in skeletal dysplasia: a review

**DOI:** 10.1152/ajpcell.00613.2023

**Published:** 2024-04-22

**Authors:** Roufaida Bouchenafa, Francesca Manuela Johnson de Sousa Brito, Katarzyna Anna Piróg

**Affiliations:** Skeletal Research Group, Biosciences Institute, Newcastle University, Newcastle upon Tyne, United Kingdom

**Keywords:** chondrocyte, kinesin, microtubules, motor proteins, skeletal dysplasia

## Abstract

Skeletal dysplasias are group of rare genetic diseases resulting from mutations in genes encoding structural proteins of the cartilage extracellular matrix (ECM), signaling molecules, transcription factors, epigenetic modifiers, and several intracellular proteins. Cell division, organelle maintenance, and intracellular transport are all orchestrated by the cytoskeleton-associated proteins, and intracellular processes affected through microtubule-associated movement are important for the function of skeletal cells. Among microtubule-associated motor proteins, kinesins in particular have been shown to play a key role in cell cycle dynamics, including chromosome segregation, mitotic spindle formation, and ciliogenesis, in addition to cargo trafficking, receptor recycling, and endocytosis. Recent studies highlight the fundamental role of kinesins in embryonic development and morphogenesis and have shown that mutations in kinesin genes lead to several skeletal dysplasias. However, many questions concerning the specific functions of kinesins and their adaptor molecules as well as specific molecular mechanisms in which the kinesin proteins are involved during skeletal development remain unanswered. Here we present a review of the skeletal dysplasias resulting from defects in kinesins and discuss the involvement of kinesin proteins in the molecular mechanisms that are active during skeletal development.

## Graphical abstract

**Figure:**
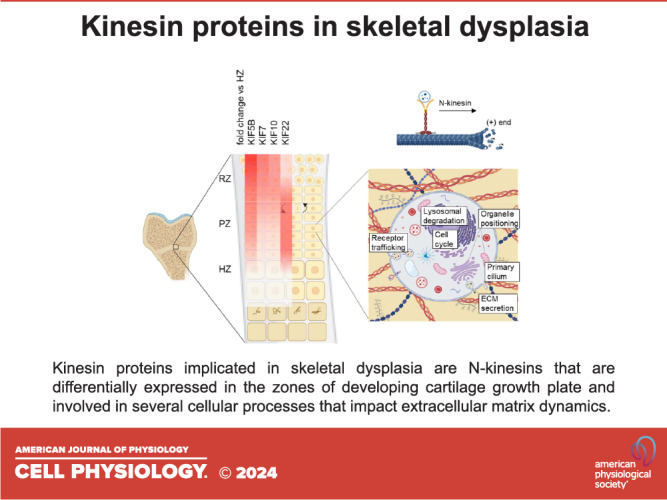

## INTRODUCTION

Skeletal dysplasias are a group of heterogeneous genetic disorders that affect skeletal growth and are characterized by often overlapping phenotypes such as dwarfism and long bone deformation and malformation (1, 2). Abnormalities in the early skeletal development, patterning, and maintenance that affect mainly bone and cartilage but often also tendons, ligaments, and skeletal muscles result in a variety of skeletal diseases with an estimated prevalence rate of 1/5,000 births worldwide (3, 4). Skeletal dysplasias are diagnosed based on radiographic, clinical, and molecular features and can be associated with further complications such as congenital cardiac defects, gastrointestinal abnormalities, endocranial, and neurological complications (2). Advances in molecular genetics and next generation sequencing have accelerated the identification of genetic variants in rare disorders of the skeleton and allowed the identification of 771 conditions associated with 552 genes (5) The molecular mechanisms underlying the skeletal dysplasias together with the identified genetic variants are detailed in the “Nosology of skeletal dysplasia,” which is regularly reviewed and updated (5). Research advances have shown that different genetic mutations acting through shared molecular pathways can cause similar skeletal phenotypes [e.g., osteogenesis imperfecta (OI) results from disruptions in genes encoding type I collagen (*COL1A1* and *COL1A2*) and genes encoding molecular chaperones (e.g., *SERPINH1* and *FKBP10*)], as well as that different skeletal dysplasias can result from mutations in the same gene (e.g., mutations in the *FGFR3* cause achondroplasia, hypochondroplasia, and thanatophoric dysplasia) (6, 7). Genes associated with skeletal dysplasias are implicated in various biological processes and encode extracellular (matrix components, matrix remodeling enzymes, growth factors) (8–10), intracellular (chaperones, transcription factors, motor molecules) (6, 11), and cell surface molecules (receptors, channel proteins) (12, 13) that are essential for bone and cartilage formation and maintenance.

### Kinesins Are Ubiquitously Expressed Microtubule-Associated Motor Proteins Involved in Multiple Cellular Processes

Kinesins are cytoskeletal motor proteins that carry cargo along the microtubules in an ATP-dependent manner (14). Kinesins were first discovered in 1985 as proteins involved in neuronal development and homeostasis, and together with dyneins they control the anterograde and retrograde axonal transport (14, 15). Since their discovery, 45 different kinesin (KIF) genes have been identified in mice and humans and were grouped into 15 classes based on sequence homology (Table 1) (78). Most kinesins function as a dimer, and their structure comprises a coiled-coil neck linker that mediates the dimerization of the two heavy chains and two light chains, which are associated by a heptad repeat regions near their N-terminus (79). The motor domain, also known as the head, binds to the microtubules and hydrolyses ATP to generate energy required for movement. The tail domain attaches to specific cargo, either directly or through association with adaptor proteins, and regulates the kinesin function (79).

**Table 1.** Classification of the kinesin proteins according to the motor domain position (16)

N-Kinesins		
Kinesin Class	Member Kinesins	Identified Roles/Processes
Kinesin 1	KIF5A	RNA transport (17), mitochondria transport (18), autophagy (19), secretory vesicle transport (20)
	KIF5B	RNA transport (17), cytokinesis (21), vesicle transport (22), peroxisome transport (23), nuclear positioning (24)
	KIF5C	RNA transport (17)
Kinesin 2	KIF3A	Embryonic cilia formation (25, 26), cilia function, anterograde IFT (27), hedgehog signaling (28)
	KIF3B	Ciliogenesis (26), hedgehog signaling (29), vesicle transport (30)
	KIF3C	Ciliogenesis (31), axonal transport (32)
	KIF17	Vesicle transport, receptor sorting (33)
Kinesin 3	KIF1A	Vesicle transport (34, 35)
	KIF1B	Mitochondria transport (36), receptor transport (37)
	KIF1C	Retrograde transport of Golgi vesicles to the ER (38)
	KIF13A	Vesicle transport, Golgi to plasma membrane transport (39)
	KIF13B	VEGFR2 trafficking from the Golgi, angiogenesis (40), secretory vesicle transport (41)
	KIF14	Cytokinesis (41)
	KIF16A	Cell division and spindle assembly (43), pericentriolar material stabilization (43)
	KIF16B	Vesicle transport, early to late endosomes (44), receptor recycling (45)
Kinesin 4	KIF4A	Chromokinesin (46), ciliogenesis (47, 48)
	KIF4B	Spindle dynamics and cytokinesis (49)
	KIF7	Primary cilium-associated hedgehog signaling (50)
	KIF21A	Microtubule dynamics (51)
	KIF21B	Microtubule dynamics, centrosome polarization (52)
	KIF27	Construction of central pair in motile cilia, ciliogenesis, and hedgehog signaling (53)
Kinesin 5	KIF11	Cytokinesis, spindle formation (54), ciliogenesis (55), translation, association of ribosomes with microtubules (56)
Kinesin 6	KIF20A	Cytokinesis (57), Golgi-derived vesicle transport (58)
	KIF20B	Cell division (59)
	KIF23	Cytokinesis (60)
Kinesin 7	KIF10	Cell division, kinetochore function (61)
Kinesin 8	KIF18A	Mitotic chromosome positioning (62)
	KIF18B	Microtubule depolymerization, cell division (63)
	KIF19A	Cilia length (64)
Kinesin 9	KIF6	Ciliogenesis, cell division (65)
	KIF9	Motile cilia function (66), podosome function (67)
Kinesin 10	KIF22	Chromokinesin (46)
Kinesin 11	KIF26A	Microtubule stability, RET signaling (68)
	KIF26B	MYH10 mediated cell-cell adhesion (69)
Kinesin 12	KIF12	Cell polarity (70)
	KIF15	Cell division (71), integrin endocytosis (72)
Kinesin 13	KIF24	Depolymerization of the centriolar microtubules (73)
Kinesin 14b	KIF25	Centrosome operation and spindle orientation (74)

M-Kinesins		
Kinesin class	Member kinesins	Identified roles/processes
Kinesin 13	KIF2A	Microtubule depolymerization, spindle assembly, and chromosome movement (75)
	KIF2B	Spindle assembly and chromosome movement (75)
	KIF2C	Microtubule depolymerization, cell division (63)

C-Kinesins		
Kinesin class	Member kinesins	Identified roles/processes
Kinesin 14a	KIFC1	Retrograde transport from Golgi apparatus to the ER (38), retrograde transport of secretory vesicles (76)
Kinesin 14b	KIFC2	Correction of the improper microtubule attachments at kinetochores (75)
	KIFC3	Maintaining the integrity of the adherent junction (77)

**Figure 1.**
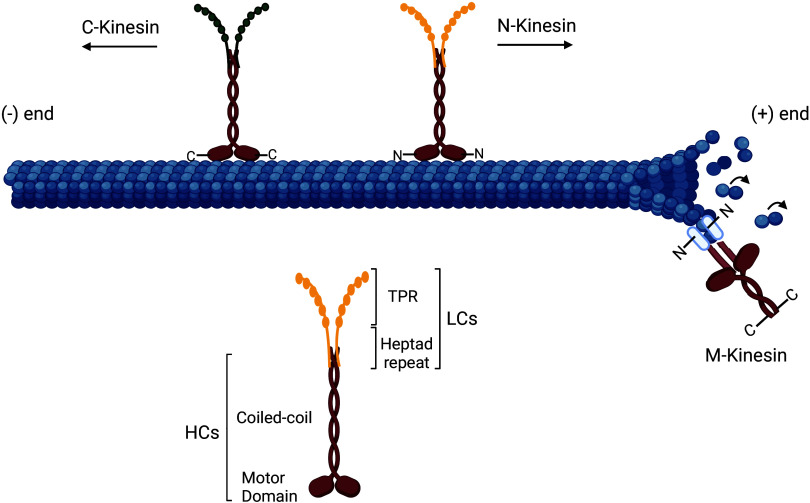
Molecular structure of kinesins. Kinesin dimers comprise two heavy chains (HCs) with a microtubule-binding site (motor domain) and a coiled-coil domain, and two light chains (LCs) comprising the tetratricopeptide repeat domain (KLC^TPR^) and a heptad repeat that associate to the coiled-coil domain of HCs (80). The position of the motor domain defines the kinesin classes: N-Kinesins have an N-terminus motor domain and drive plus-ended microtubule movement, M-Kinesins have their motor domain in the middle and are MT depolymerizers, and C-kinesin have a carboxy (C)-terminal motor domain and mediate minus-end-directed movement (81, 82). Created with BioRender.com.

The kinesin motor domain is located either in the amino (N)-terminus, middle of the protein, or in the carboxy (C)-terminus. Based on this, kinesins are subdivided into three classes: N-kinesins that are plus-end-directed motor protein, M-kinesins that act as microtubule depolymerizers, and C-kinesins that mediate minus-end-directed movement (Fig. 1) (81, 82). The motor domains present a high sequence homology in all kinesin superfamily and are highly conserved across a number of species (83). Kinesin microtubule binding and movement are regulated by various intramolecular and intermolecular interactions; for example, Kinesin-1 molecules are autoinhibited by the kinesin head and tail interactions, whereas Kinesin-3 molecules autoinhibition is via interaction of the coiled-coil domain with the motor head (79, 83, 84). The diversity of the kinesin light chains permits the recognition and transport of multiple cargos (85). There are four human kinesin light chains (KLCs, KLC1, KLC2, KLC3, and KLC4), and their complexity can be further enhanced by alternative splicing, with multiple splice variants allowing specific cargo binding (83). Cargos can bind directly to the kinesin heavy chains or attach to the tetratricopeptide (TRP) repeat domain in the kinesin light chain (KLC) (86). The TRP repeats are composed of multiple tandem repeats of 34 degenerate amino acids forming a helix-turn-helix structural motif able to mediate protein-protein interactions (80). Activation of kinesins is by effector proteins binding to the kinesin heavy chain or the TRPs on the light chain and breaking or weakening the intramolecular interaction (86–88).

Kinesin proteins are essential for neurodevelopment and have been implicated in cancer development and metastasis. They are involved in several key molecular processes, such as the transport of organelles, vesicles, membrane-bound organelles, nucleic acids, and protein complexes, and in the regulation of cell cycle progression, mitotic spindle formation, and movement of chromosomes during cell division (78). Kinesin family members also participate in the regulation of microtubule dynamics and were recently discovered to act as depolymerizers (KIF2A and KIF2C) and microtubule stabilizers (KIF26A and KIF21A), further emphasizing their importance for cellular morphogenesis, neuronal and axonal morphology, and ciliogenesis (Table 1) (89).

### Several Kinesins Are Implicated in Skeletal Development and Disease

**Figure 2.**
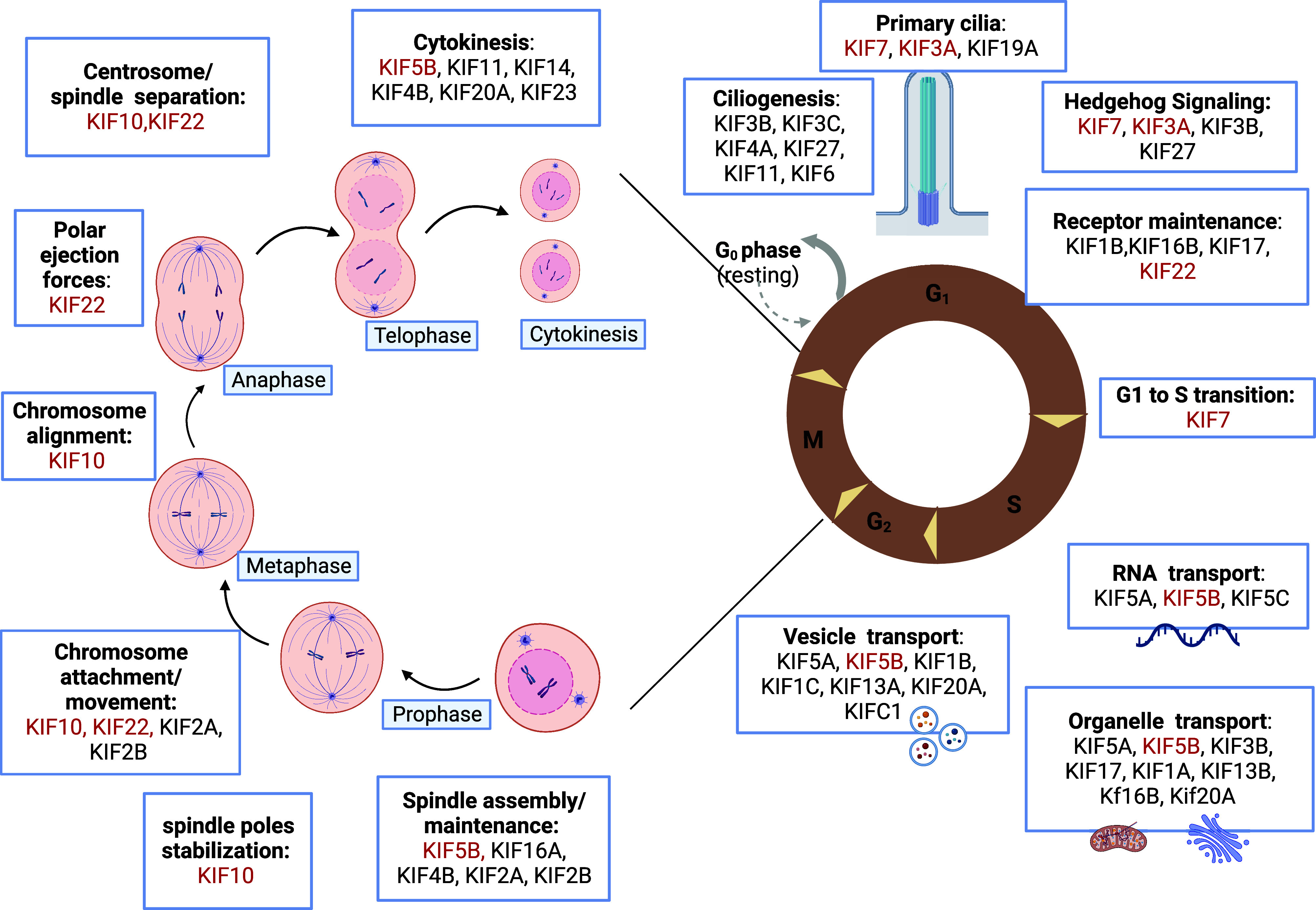
Kinesins play a role in multiple cell processes. Kinesins ensure specific functions during different phases of cell division, mediating chromosome movement and alignment, spindle formation, and regulating microtubule-kinetochore interaction. They are also involved in the maintenance of the primary cilia structure and function, and drive intracellular transport of organelles, vesicles, receptors, and RNA along microtubules. The kinesins implicated in skeletal dysplasia have been highlighted in red. Created with BioRender.com.

**Figure 3.**
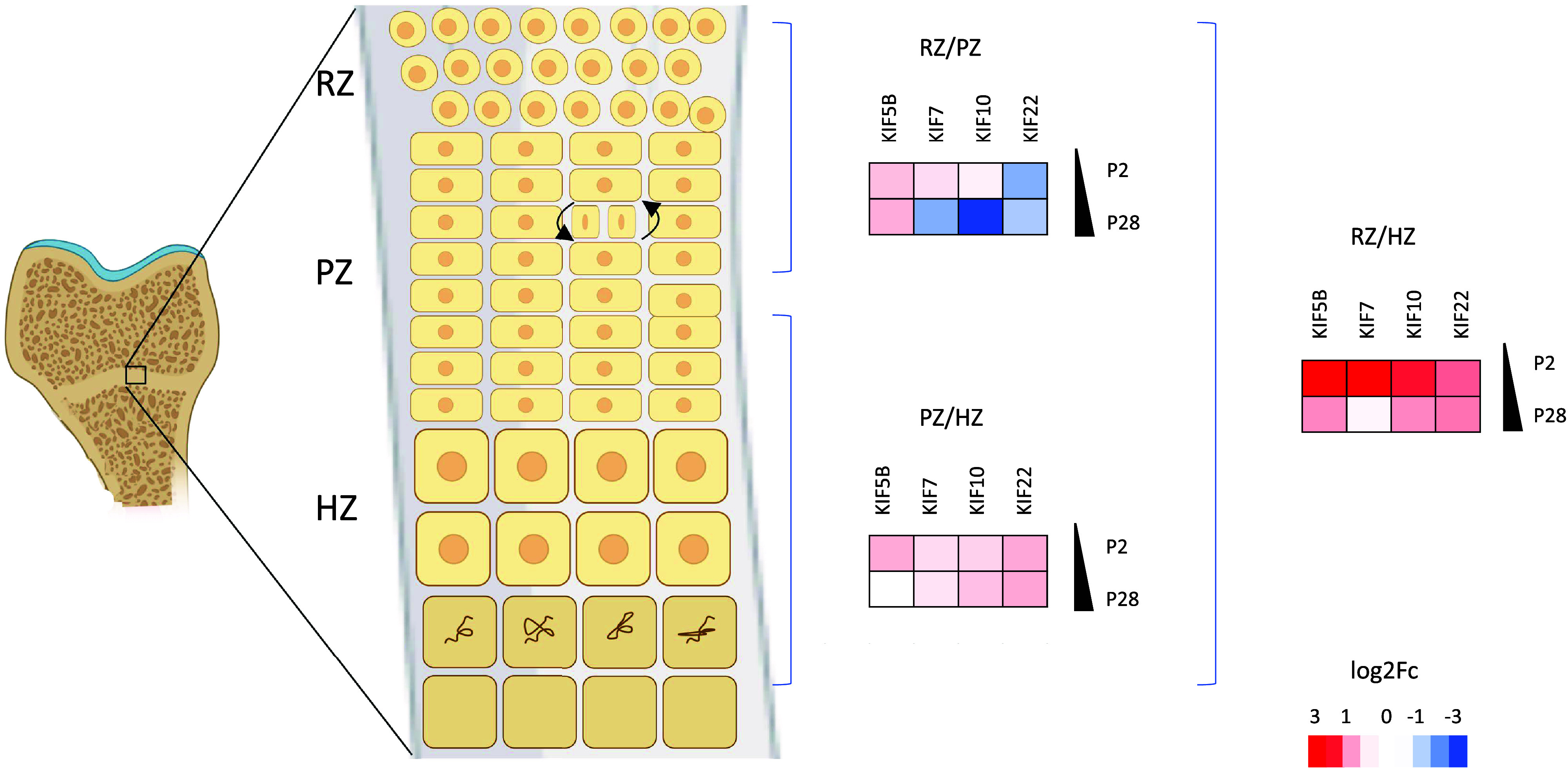
KIF5B, KIF7, KIF10, and KIF22 are differentially expressed in the zones of murine epiphyseal cartilage during development. Fold change data were obtained from SkeletalVis (106), and heat maps showing differential expression (log_2_FC) were generated. Key: HZ, hypertrophic zone; PZ, proliferative zone; P2, postnatal day 2; P28, postnatal day 28; RZ, resting zone. Created with BioRender.com.

**Figure 4.**
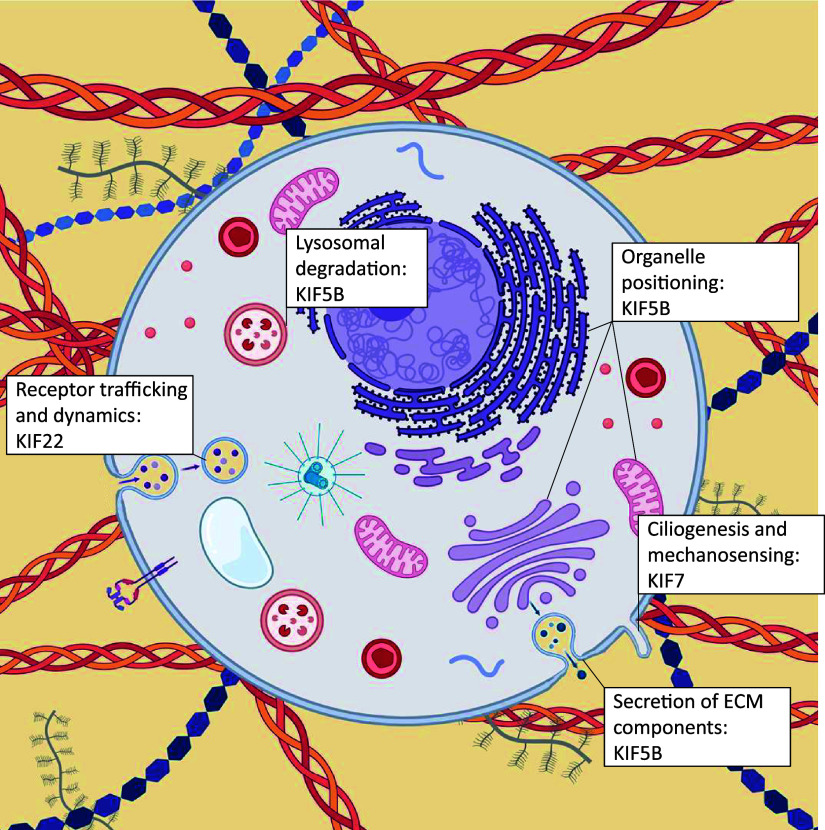
Kinesin motor proteins mediate ECM-related processes. Kinesin molecules implicated in skeletal dysplasia play roles in organelle positioning (including the endoplasmic reticulum and Golgi apparatus) and receptor recycling, and contribute to the intracellular transport of vesicles containing ECM components. Kinesins also play roles in ciliogenesis and mechanosensing. ECM, extracellular matrix. Created with BioRender.com.

Kinesins play a pivotal role during early development and organogenesis. They are essential for neuronal development and axonal transport and play important roles in cell survival. Mutations in several kinesin genes affect neurodevelopment and result in secondary microcephaly, and in several mouse models the loss of kinesin function led to embryonic or perinatal lethality (KIF1A, KIF1B, KIF2A, KIF3A, KIF3B, KIF5A, KIF5B, KIF7, KIF10, KIF11, KIF18A, KIF20A, KIF20B, KIF21, and KIF26B) (90–92). Interestingly, mutations in several kinesin genes have also been specifically shown to have an impact on the development of the vertebral column and the appendicular skeleton and lead to multiple malformations, reduced bone formation, early onset joint degeneration, and multiple joint dislocations. Skeleton formation, maintenance, and homeostasis are tightly regulated by numerous molecular mechanisms in which kinesins play a key role; and mutations in four kinesin genes are associated with specific skeletal dysplasia (Table 2), through a variety of mechanisms (Fig. 2). Differential spatial and temporal gene expression was detected for *Kif5b*, *Kif7*, *Kif10*, and *Kif22* in the murine tibial growth plate cartilage across the growth plate zones and with age (Fig. 3), highlighting their importance in the resting and proliferative zone homeostasis. Disruption of KIF5B has been shown to affect cytokinesis of proliferative chondrocytes and result in growth plate disorganization and skeletal abnormalities (21). HH signaling pathways are important for the maintenance of growth plate and skeletal patterning, and mutations in KIF7 impair HH signaling in the growth plate (50). In addition, KIF7 plays crucial role in cilia length regulation (94). Chondrocytes are highly mechanoresponsive and defects in primary cilia homeostasis have been shown to compromise cartilage mechanosensing and lead to skeletal dysplasia. Moreover, kinesin proteins are essential for cell division, and mutations in kinesins tend to strongly affect mitosis in skeletal cells. In the growth plate, chondrocytes undergo constant division and can be affected by mutations in the chromokinesin proteins (e.g., KID/KIF22 and kinetochore kinesin CENPE) through mechanisms such as aberrant chromosome attachment and movement on the spindle microtubules, chromosome misalignment on equatorial plate, and disrupted centrosome separation (95). Furthermore, kinesin proteins implicated in skeletal dysplasia are involved in organelle positioning and function and in vesicular transport, which can impact on ECM-related processes, and consequently lead to abnormal ECM organization and skeletal defects (Fig. 4) (20).

**Table 2.** Skeletal dysplasias resulting from mutations in kinesin genes (5)

Gene	Inheritance	Disorder	MIM No.	Skeletal Features	Complications	Disease Mechanism
*KIF5B*	AD	Kyphomelic dysplasia with facial dysmorphism (21, 107, 110)	211350	Short stature, vertebrae deformity, narrow chest, bowed legs, joint stiffness, micromelia, distinctive facial features, and micrognathia	Neonatal respiratory distress	Impaired mitochondria and lysosome trafficking, disruption of autophagy, cytokinesis defects, proliferative chondrocyte apoptosis, disrupted perichondral ossification
*KIF7*	AR	Acrocallosal syndrome (ACLS) (115)	200990	Macrocephaly, facial dysmorphism, brain abnormalities	Mental retardation	Disruption of HH signaling, cilia length misregulation, proliferative chondrocyte apoptosis, and expansion of the hypertrophic zone
	AR	Hydrolethalus syndrome (HLS2) (94)	614120	Hydrocephaly, postaxial polydactyly of the upper limbs, and pre- or postaxial polydactyly of the lower limbs and hallux duplication	Widened ventricles and midbrain-hindbrain malformation	
	AR	Al-Gazali-Bakalinova syndrome (AGBK) (114)	607131	Macrocephaly multiple epiphyseal dysplasia, and distinctive faces	Agenesis of the corpus callosum and frontotemporal brain atrophy	
*KIF10*	AR	Microcephalic osteodysplastic primordial dwarfism (61, 101, 102, 126)	616051	Microcephaly, short stature, metaphyseal sclerosis, structural brain malformations, mild micrognathia, short metacarpals, and osteopenia	Partial agenesis of the corpus callosum and cerebellar hypoplasia, cardio-myopathy	Chromosome mis-alignment, spindle defects, and arrest of G2-M phase
*KIF22*	AD	SEMD with joint laxity (Hall type or leptodactylic type) (103, 129, 131)	603546	Short stature, progressive knee malalignment, slender metacarpals/metatarsals, mild spinal deformity, and early onset osteoarthritis	Ligamentous laxity, multiple dislocations	Chromosome segregation defects, lack in chromosome compaction, and disrupted cytokinesis

### Loss of Function of KIF5B Affects Cell Polarity, Cytokinesis, and Lysosomal Function and Results in Kyphomelic Dysplasia with Facial Dysmorphism

Kyphomelic dysplasia with facial dysmorphism is an autosomal recessive skeletal disorder characterized by a disproportionately short stature with a short narrow chest, shortening and bending (bowing) of the long bones, flared irregular metaphyses, and characteristic facial features (depressed nasal bridge, hypertelorism, micrognathia, and mid face hypoplasia) (107). Three *de novo* heterozygous variants in *KIF5B* encoding kinesin heavy chain have recently been reported, affecting the highly conserved amino acids near the ATP binding site in the catalytic motor domain of KIF5B (107). KIF5B is a member of the Kinesin-1 family, widely expressed in the human body with relative abundance in the retina, skeletal muscle, and central nervous system (108, 109).

In zebrafish, mutations in *Kif5B* (*Kif5Blof*) impaired lysosomal function and consequently led to a deregulation of autophagy (110). Moreover, *Kif5B* loss of function induced chondrocyte apoptosis and led to a loss of flattened appearance of the proliferative chondrocytes, accumulation of abnormally rounded chondrocytes with impaired polarization, loss of hypertrophic chondrocytes, and disrupted perichondral ossification (110). Centrosomes in the mutant cells were abnormally orientated, suggesting the loss of polarization of the microtubule-organizing center (MTOC). A chondrocyte-specific knockout mouse model of *Kif5b* (*Col2*cre; *Kif5b*^fl/−^ mice) provided further insights into the intracellular role of KIF5B in chondrocytes. *Kif5b* mutant mice displayed short stature with restricted growth of spine vertebrae and long bones (21). Mutant growth plate showed disrupted cartilage characterized by disorganized columnar structure, cellular misorientation, abnormal apoptosis of proliferative chondrocytes, and a decrease in the number of hypertrophic chondrocytes (24). Monitoring cell division of isolated mutant chondrocytes demonstrated defects in cytokinesis characterized by a delay in cell abscission or failure in forming daughter cells due to back fusion during abscission of the primary chondrocytes. KIF5B has been shown to concentrate in the central spindle of primary chondrocytes, and the disruption of the mitotic spindle in the KIF5B null chondrocytes outlines its role in the organization and maintenance of the central spindle during chondrocyte cytokinesis. Deletion of KIF5B in all mouse tissues results in embryonic lethality and severe growth retardation (111). Interestingly, the extraembryonic cells used to study the intracellular mechanisms affected by the global *Kif5B* deletion showed abnormally clustered mitochondria in the perinuclear region of cells and impaired lysosomal dispersion. The mitochondria phenotype was rescued by an exogenous expression of *Kif5B*, highlighting a role of KIF5B in both mitochondria and lysosome trafficking and positioning (111). *Kif5blof* zebrafish mutants also showed abnormal mitochondria and ER localization (110). The impaired lysosomal function reported in the *Kif5Blof* zebrafish mutants correlated with decreased mTOR pathway activation that may have contributed to the decrease in the number of hypertrophic chondrocytes and suggested a role for KIF5B in regulating autophagy. Moreover, a heterozygous mutation in *KIF5B* was identified in four patients with osteogenesis imperfecta (OI) and analysis of primary patient fibroblasts showed an impaired intracellular transport of mitochondria and abnormal Golgi positioning and resulted in a downregulation of mTOR signaling supporting the implication of KIF5B in regulation of autophagy (98). In addition, Kinesin-1 and -3 proteins have been shown to drive lysosome movement along different microtubule tracks and particularly KIF5B selectively recruits lysosomes to the microtubules (97). KIF5B has also been shown to be important for the initial formation of the autophagosome in the HeLa cells (97). Interestingly, KIF5B has been implicated in the delivery of matrix metalloproteinases to the cell surface in macrophages and cancer cells (96, 112), highlighting the diversity of individual kinesin functions and potential additional roles for this kinesin in skeletal development and homeostasis. For example, it has been shown that KIF5B is important for MMP14 (also implicated in cartilage resorption and ossification) trafficking in macrophages (113). KIF5B has also been shown to be involved in the intracellular trafficking of secretory vesicles containing extracellular matrix components and colocalized with collagen I-containing vesicles in the human pleural mesothelial cells, with KIF5B deletion resulting in reduced type I collagen secretion (20). Type II collagen secretion was also affected in *Kif5blof* zebrafish mutants (110). KIF5B may therefore contribute to the extracellular matrix production and organization by playing a role in transporting secretory vesicles. Taken together, KIF5B is a major contributor to the intracellular trafficking of various organelles (mitochondria, lysosomes, and secretory vesicles) that are implicated in extracellular matrix synthesis and skeletal development, as well as playing a role in cell division where it contributes to the organization and maintenance of the central spindle during cytokinesis.

### Allelic Series of *KIF7* Mutations Lead to Ciliopathy-Related Skeletal Disorders

Genome-wide linkage analysis and gene sequencing of individuals manifesting with several skeletal disorders identified homozygous mutations in *KIF7* in three autosomal recessive lethal disorders commonly characterized by dysmorphic features (macrocephaly, hydrocephaly, or anencephaly) and skeletal dysplasia (114). Hydrolethalus syndrome manifests with hydrocephaly, micrognathia, postaxial polydactyly of the upper limbs, and pre- or postaxial polydactyly of the lower limbs and hallux duplication and is associated with a homozygous deletion in the KIF7 coiled-coil domain (94). Al-Gazali-Bakalinova syndrome resulting from the missense mutations in *KIF7* is characterized by macrocephaly, multiple epiphyseal dysplasia, *genu valgum* (knock knees), clinodactyly, short neck, *pectus excavatum*, and distinctive facial features including hypertelorism and frontal bossing (114). Acrocallosal syndrome (ACLS) results from nonsense or frameshift *KIF7* mutations affecting the motor domain or the Gli binding site and presents with macrocephaly, mental retardation, polydactyly, hallux duplication, and characteristic facial features including hypertelorism and prominent forehead (115). Novel heterozygous missense mutations have also been identified in *KIF7* and shown to be associated with primary cilia defects that lead to ciliopathies, in particular the Bardet-Biedl syndrome (94). Homozygous *KIF7* mutations have also been reported in Joubert syndrome; a ciliopathy with phenotype overlapping with the ACL syndrome (116).

HH signaling plays a key role in tissue development and cellular homeostasis, including skeletal patterning, development, and growth; it is highly associated with primary cilia (cell surface organelles involved in regulation of cell polarity and mechanosensing) and implicated in pathobiology of ciliopathies (117). Interestingly, chondrocytes from mice null for KIF3A, a subunit of the Kinesin-2 motor complex required for intraflagellar transport, showed loss of primary cilia, reduced proliferation, defective cell rotation, and accelerated differentiation, resulting in disrupted columnar organization in the growth plate and postnatal dwarfism (118). It has been shown that the mutant KIF7 also results in defects in primary cilia formation and induces abnormal centrosomal duplication and fragmentation of the Golgi network, indicating that KIF7 can potentially participate in microtubule stability and growth direction (119). Analysis of fibroblasts from patients with ACLS showed longer primary cilia without a disruption in the cilia components, suggesting a potential role of KIF7 in cilia length regulation (94). KIF7 was found to localize to the primary cilia tips (the microtubule plus end) in Sonic Hedgehog (SHH)-stimulated cells where it regulates the cilia length by promoting microtubule catastrophe and limiting microtubule polymerization (50). Further research showed that KIF7 mutations cause ciliopathies through dysregulation of the Hedgehog (HH) signaling pathway components and that KIF7 is a key mediator in the HH signaling pathway through the regulation of Gli transcription factor targets (50). *Kif7*^−/−^ mice, a representative model of ACLS, exhibited most of the features observed in the patients with ACLS and were used to investigate the molecular pathology of the disorder (120). In this model, loss of function of KIF7 resulted in aberrant Gli3 activity and overexpression of SHH signaling, suggesting that KIF7 is essential to regulate Gli3 repressive activity during embryonic development. SHH regulates the anterior-posterior patterning of the limbs, and Indian Hedgehog (IHH) signaling is important for regulation of the chondrocyte proliferation and differentiation in the cartilage growth plate (121). Specifically, IHH is expressed by the prehypertrophic and hypertrophic chondrocytes, regulates the rate of hypertrophic differentiation, and stimulates the expression of parathyroid hormone-related protein (PTHrP) in the resting chondrocytes, which in turn negatively regulates hypertrophy (122). It is also important in the perichondrium where it regulates the osteoblast differentiation and bone formation (123). Disruption of IHH and its associated Gli zinc-finger proteins (Gli1–Gli3) renders proliferative chondrocytes unable to initiate the hypertrophic differentiation process and has a high impact on chondrogenesis and bone formation (123, 124). Deletion of IHH in mice affects osteoblast development during endochondral ossification, reduces chondrocyte proliferation, and results in expanded hypertrophic zone in the growth plate (122). KIF7 has been shown to be a major mediator of IHH signaling, regulating Gli transcription factors together with SUFU (99, 100). During development, KIF7 downregulates SUFU to promote the IHH pathway. In the absence of SUFU, it represses IHH signaling by inhibiting Gli-mediated transcription, reducing HH pathway activity, decreasing chondrocyte proliferation, and expanding the hypertrophic zone. Moreover, studies using several SHH-signaling pathway mutant mouse strains crossed with the *Kif7^Maki/Maki^* mouse model developed through ENU mutagenesis confirmed that KIF7 acts downstream of Smoothened (Smo) and upstream of Gli, and its activity is dependent on the presence of the primary cilia (125). Interestingly, differential expression of KIF7 was demonstrated by qRT-PCR on microdissected sections of wild-type mouse growth plate and shown that KIF7 expression is high in the articular and resting chondrocytes and significantly decreased in proliferating, prehypertrophic, and hypertrophic chondrocyte, similar to the SUFU and PTHrP expression (121). In summary, KIF7 is essential for the regulation of chondrocyte proliferation and differentiation, controls cilia length during ciliogenesis by promoting microtubule catastrophe, is a key mediator of HH signaling, and its deletion or disruption results in skeletal defects and ciliopathies.

### Compound Heterozygous Mutations in KIF10 Disrupt Cell Division and Are Associated with Primary Microcephalic Osteodysplastic Primordial Dwarfism

Microcephalic osteodysplastic primordial dwarfism (MCPH13) is an autosomal recessive skeletal disorder characterized by limited growth, small hands and metaphyseal sclerosis, mild micrognathia, short metacarpals, and osteopenia (61). Whole genome sequencing has identified two variants of compound heterozygous mutation of *KIF10* (c.2797G>A: p.D933N and c.4063A>G: p.K1355E) in individuals with MCPH13 as causative locus of this disorder.

KIF10 (also known as centromere-associated protein E, or CENPE) is a kinetochore-associated kinesin-like motor protein (101). CENPE gene is evolutionary conserved in humans, mice, and zebrafish. During cell division, kinesin proteins participate in the separation of centrosomes to achieve spindle bipolarity and facilitate chromosome congression to the metaphase plate through diverse mechanical activities; transducing regulatory signals, modulating microtubule ends, and directing movements (plus-end, minus-end, or bipolar plus-end motility) (61). Kinesins involved in the kinetochore-microtubule interactions are also essential for chromosome alignment (95). They mediate chromosome attachment and movement along spindle microtubules, generate tension across aligned chromosomes, maintain their alignment, and stabilize spindle poles (95). HeLa cells with depleted CENPE showed chromosome misalignment at metaphase and abnormal spindle microtubules. Interestingly, the ratio of cells in the G2-M phase of the cell cycle was significantly decreased indicating the arrest of the G2-M phase (101, 102). CENPE null mice resulting from a heterozygous cross die *in utero* and immunofluorescence analysis of in vitro culture of early-stage embryos showed severe abnormal growth, inner cell mass growth retardation, aberrant mitosis, and chromosome misalignment (126). Moreover, examination of CENPE-deficient fibroblasts revealed a significant decrease in the number of kinetochore-associated microtubules, misposition of the chromosome centromeres, and defects or failure in chromosome alignment, highlighting that KIF10 is essential for kinetochore-microtubule interaction stability and for chromosome stability, alignment, attachment to the spindle, and for mitotic check-point activation (126). Furthermore, inhibition of the ATP binding sites of the CENPE motor domain in zebrafish using a small-molecular inhibitor (GSK923295), used as a chemotherapeutic to disrupt mitosis in cancer cells, resulted in severe developmental abnormalities characterized by head malformation, degenerative notochord, smaller lens, and small organs. Analysis of the early embryogenesis stage showed asymmetric cell division of the zygotes and disruption of cell cycle with defects in cell migration during gastrulation, suggesting the role of CENPE in the regulation of cell division and organogenesis (102). In mice, CENPE inhibition using the same molecular inhibitor (GSK923295) significantly reduced the weight of the liver, heart, and kidney, and mutant liver cells showed an abnormal cell morphology and arrest of cell cycle at metaphase, confirming the critical role of CENPE in early development of the organs (102). In contrast, CENPE-deficient liver cells from mice with a conditional disruption of CENPE injured by carbon tetrachloride injection to induce chemical damage manifested abnormal chromosome positioning and unstable microtubules-kinetochores interactions but were able to evade mitotic arrest and consequently regenerate at normal rate and recover their function, suggesting that CENPE plays a critical role in early development but not in late stages of organogenesis (126). Altogether, these studies demonstrate the role of KIF10 in early embryogenesis and early organ development, as key regulator of cell cycle progression and key mediator of cell division, essential for chromosomes alignment and microtubule spindle stability.

### Gain of Function Mutations in the Head and Tail Domain of KIF22 Result in Spondyloepimetaphyseal Dysplasia with Joint Laxity

Spondyloepimetaphyseal dysplasia with joint laxity type 2 (SEMDJL-leptodactylic type or SEMDJL2) is an autosomal dominant skeletal disorder characterized by short stature, limb malalignment (*genu valgum* and/or *varum*), ligamentous laxity, and mild spinal deformity without cognitive impairment (103). Individuals with SEMDJL2 present with delayed epiphyseal ossification leading to epiphyseal dysplasia, striated metaphyses, and precocious osteoarthritis (127). Other common features include joint laxity, slender metacarpals, constricted femur neck, and progressive scoliosis, all together presenting a premature skeletal aging phenotype (104). The molecular pathogenesis of the SEMDJL-leptodactylic type was thought to be related to ECM abnormalities since the disorder is characterized by highly abnormal tendon collagen fibrils in the affected ligaments and early onset joint degeneration. This hypothesis was encouraged by the observation of phenotypic overlap with skeletal disorders in which either collagen type II or cartilage oligomeric matrix protein (COMP) is affected (spondyloepiphyseal dysplasia congenita and pseudoachondroplasia, respectively) (128). However, the disease-causing mutations in patients with SEMDJL2 remained obscure until the whole genome sequencing of individuals with lepto-SEMDJL in 2011 identified *KIF22* mutations as the main cause of the disorder (104).

Analysis of early mouse embryos showed KIF22 attachment to the chromosomes during anaphase/telophase in a chromosome- and microtubule-binding-dependent manner and revealed that KIF22 deficiency results in malformation of nuclear blastomeres and affects cell division. KIF22 deletion affected early embryonic development of pups derived from intercrosses of *Kif22*^+/−^ with 50% of KIF22 null embryos dying by E9.5 (129). Time-lapse imaging of chromosomes in *Kif22*^−/−^ zygotes showed less chromosome compaction during anaphase/telophase and 40% of micro- or multinucleation at the 1–8 cell stage. Interestingly, KIF22 deficiency did not severely affect meiosis and the post morula mitosis (129). The *Kif22*^−/−^ pups that survived and developed to adulthood without an overt phenotype were those that passed the 4- to 8-cell stage without multinucleated blastomeres. Four of the discovered SEMDJL2 mutations affect the KIF22 motor domain and result in the substitution of two highly conserved amino acids in the α2 helix of the KIF22 motor domain near the ATP binding site [Proline 148 (P148S, P148L) and Arginine 149 (R149Q, R149L)] and consequently interfere with the ATP binding (104, 105, 128). The mutations were exclusively identified in individuals with SEMDJL2 and had largely tissue-specific effects particularly in the connective tissues, despite the *KIF22* mRNA expression detected in various human somatic tissues (130). An additional SEMDJL2 mutation was recently identified in the KIF22 coiled-coil domain in the kinesin tail, with a highly conserved Valine 475 residue substituted by Glycine, and suggesting that a second microtubule binding site may be present in the tail of KIF22 that is as important as the head motor domain (131). It has been suggested that mutations in either the tail or motor domain of KIF22 can impede the interaction between these domains and subsequently prevent KIF22 inactivation. Moreover, phosphorylation of two residues in KIF22 (T158 and T463) that are in the vicinity of the mutation sites disrupted KIF22 inactivation and hampered chromosome segregation, which highlighted the essential role of T463 and T158 dephosphorylation in promoting the head-tail interaction to inactivate KIF22. Interestingly, KIF22 with SEMDJL2 mutations was able to attach to both chromosome arms and spindle microtubules in Kyoto-Hela cells (131). In normal conditions, KIF22 inactivation during anaphase produces ejection forces to facilitate chromosome movement of the spindle poles (132, 133). KIF22 tail mutations disrupted the chromosome segregation during anaphase due to its hyperactivation and generation of aberrant polar ejection forces, which limit chromosome arm movement, disrupt spindle separation, and lead to chromosome misplacement in the spindle (131). Daughter cells resulting from the HeLa cells overexpressing recombinant mutant KIF22 were multinucleated and exhibited lack of anaphase chromosome mass compaction along the spindle axis (129). Proliferation of cells expressing mutant KIF22 was significantly reduced due to the nuclear deformity of daughter cells and inhibition of cytokinesis. Moreover, activation of the motor domain resulted in the same phenotypes as observed for the KIF22 SEMDJL2 mutations, suggesting that the motor domain potentially regulates KIF22 activity at the metaphase to anaphase transition. Motor domain deletion in NIH 3T3 cells led to KIF22 mislocalization to anaphase chromosomes and KIF22 tail-mutant was strictly localized to the kinetochore microtubules (129). Thus, both the motor domain and the DNA binding tail domain are important for Kinesin-10 spindle localization and function during cell division.

These findings highlight the essential role of KIF22 in chromosome localization and a role in preventing cell multinucleation by mediating anaphase/telophase chromosome compaction. However, further studies are required to explain the mechanism by which KIF22 contributes to chromosome compaction, as well as the exclusively musculoskeletal phenotype of the patients with SEMDJL2 (129, 134). Analysis of KIF22 gene expression in wild-type mouse growth plate revealed its overexpression in the proliferative zone, which supports its involvement in chondrocyte proliferation (128). Interestingly, recent studies in lung carcinoma (cells and tissues from xenografts) have revealed interaction of KIF22 with cell surface receptors such as EGFR and CAR (important for the cell-cell adhesion in various tumors) and suggested its role in membrane receptor trafficking and dynamics. These data highlight the potential new roles of the chromokinesins in intracellular transport of cargo in addition to their fundamental roles in chromosome movement during cell division, which requires further investigation in the context of skeletal dysplasia (135, 136).

## SUMMARY

Mutations in KIF5B, KIF7, KIF10, and KIF22 kinesin proteins lead to skeletal dysplasia with various severity of clinical features and overlapping phenotypes. Several mutations in the kinesin motor domain, tail, or coiled-coil domain disrupt skeletal development, causing musculoskeletal complications. Interestingly, defects in kinesins have tissue-specific effects, can present with variable severity, and act through various mechanisms during different stages of embryonic development. One possible explanation as to why mutations in several kinesins manifest with pronounced skeletal phenotypes is that the expression of kinesins varies throughout tissues, indicating their varying relevance during tissue formation and, subsequently, their distinct impacts in case of disruption and dysfunction. In addition, kinesins can be differentially expressed (both spatially and temporally) within the same tissue, for example the cartilage growth plate. Moreover, several kinesins are present in different isoforms, and it is likely that some isoforms can be tissue specific. Several studies highlighted the role of kinesins in the regulation of chondrocyte proliferation and differentiation through a variety of molecular mechanisms, including regulation of signaling pathways and control of cytokinesis. Moreover, motor proteins mediate primary cilia structure and function, and their disruptions can lead to skeletal ciliopathies. Kinesins are also key modulators of cell cycle progression mediating chromosome congression and microtubule-kinetochore stability in addition to their roles in endocytosis and receptor recycling, autophagy, and intracellular trafficking of membrane-bound organelles. Although the coordination and regulation of kinesin expression in skeletal cells and the specific mechanisms in which they are involved in skeletal development and disease are not well understood, the diversity of the microtubule structures and functions together with the complexity of the regulation of the kinesins’ gene expression, splicing, and interaction with other cargo and associated partners encourages further research studies to decipher their role in development.

## GRANTS

This work was supported by HORIZON EUROPE Marie Sklodowska-Curie Actions (MSCA) Grant 101072766.

## DISCLOSURES

No conflicts of interest, financial or otherwise, are declared by the authors.

## AUTHOR CONTRIBUTIONS

R.B. prepared figures; R.B. drafted manuscript; F.M.d.S.B. and K.A.P. edited and revised manuscript; K.A.P. approved final version of manuscript.
